# OP27 Latin American Collaboration In Health Technology Assessment Of Medical Devices: Methods And Practices

**DOI:** 10.1017/S0266462325100858

**Published:** 2025-12-29

**Authors:** Victoria Hurtado-Meneses, Verónica Gallegos-Rivero

## Abstract

**Introduction:**

Medical devices represent a challenge for health systems due to their complexity in decision-making analysis. In 2019, as part of the initiatives of the Health Technology Assessment Network of the Americas (RedETSA), the Working Group on Health Technology Assessment (HTA) for Medical Devices (MD) was established to strengthen capacities, explore methodologies, and share experiences among member countries. This group comprises 16 institutions from nine countries and serves as a collaborative platform to identify and address common areas of interest for the region.

**Methods:**

Since its formation, the working group has met quarterly virtually. Progress is presented at the annual RedETSA meeting, and a common agenda has been established and coordinated by two countries. Initially, the group conducted a systematic literature review (SLR) to identify needs and new challenges in HTA for MD. Subsequently, the group collaborated on a critical analysis of the World Health Organization’s HTA of MD draft. The latest initiative started with periodic presentations on the status of HTA in MD by each member.

**Results:**

There is significant regional interest in addressing MD. The SLR results highlighted the need to establish methodologies across clinical, economic, organizational, ethical, and social dimensions. Collaboration among members has been vital in providing regional perspectives on technical documents and discovering methodologies developed in some countries that offer advancements and examples. Despite common needs, maintaining collaboration is challenging due to a lack of exclusive resources, staff turnover, and diverse health system structures. Coordinated efforts are essential to develop new methodologies to aid decision-making in health systems with limited resources.

**Conclusions:**

Despite common needs, maintaining collaboration is challenging due to limited dedicated resources, staff turnover in member countries, and diversity in health system structures. This initiative aimed to strengthen HTA processes for medical devices in Latin America through collaborative efforts, innovative methodologies, and shared experiences, ultimately ensuring the effective and safe implementation of medical technologies in Latin American health systems.

